# Hydrographic Processes Driven by Seasonal Monsoon System Affect Siphonophore Assemblages in Tropical-Subtropical Waters (Western North Pacific Ocean)

**DOI:** 10.1371/journal.pone.0100085

**Published:** 2014-06-16

**Authors:** Wen-Tseng Lo, Shwu-Feng Yu, Hung-Yen Hsieh

**Affiliations:** 1 Institute of Marine Biotechnology and Resources, National Sun Yat-sen University, Kaohsiung, Taiwan; 2 Institute of Marine Biodiversity and Evolutionary Biology, National Dong Hwa University, Checheng, Pingtung, Taiwan; 3 National Museum of Marine Biology and Aquarium, Checheng, Pingtung, Taiwan; University of Sydney, Australia

## Abstract

This work is a part of the Taiwan Cooperative Oceanic Fisheries Investigation, the first large scale hydrographic and plankton survey around Taiwan (21–26°N, 119–123°E). The present study examined the influence of hydrodynamic and biological variables driven by monsoon system on the siphonophore assemblages through an annual cycle in 2004. Calycophorans, namely *Chelophyes appendiculata*, *Diphyes chamissonis*, *Lensia subtiloides*, *Bassia bassensis*, and *Muggiaea atlantica*, were the most dominant siphonophore species. Maximum abundance of these dominant species generally occurred during the warm period (May and August), while *M. atlantica* had a significantly peak abundance in February. Although no apparently temporal difference in siphonophore abundance was observed in the study, siphonophore assemblage was more diverse in August than in other sampling times. Result of a cluster analysis indicated that assemblage structure of siphonophores in the waters around Taiwan varied at temporal and spatial scales during the sampling period. The intrusions of the Kuroshio Branch Current and China Coastal Current to the study area play an important role on the transportation of siphonophores. Also, the distribution of siphonophore assemblage was closely related to the hydrographic characteristics, with temperature, chlorophyll *a* concentration, and zooplankton abundance being the major environmental factors affecting the spatio-temporal variability of siphonophores. This study contributes substantially to the new knowledge of the siphonophore assemblage in the tropical-temperate waters of Taiwan.

## Introduction

Siphonophores, a group of complex colonial organisms, are widespread in the marine pelagic realm [1], [2]. These planktonic cnidarians are absolute carnivores, preying mainly on copepod crustaceans, and sometimes on fish larvae and young fishes [2], [3], [4]. Therefore, they usually serve as an important link between zooplankton and higher trophic levels in the pelagic food webs [4], [5]. The seasonal distribution and abundance of siphonophores are primarily governed by environmental factors controlling their reproductive cycle [6], [7]. When environmental conditions are favorable, siphonophores can reproduce rapidly by asexual reproductive processes and may at times become the most abundant non-crustacean invertebrate predators in the sea [8], [9], [10]. To understand their role in planktonic food webs naturally requires information on their biology and ecology, including temporal and spatial aspects.

The waters around Taiwan are mainly dominated by four oceanic currents: China Coastal Current (CCC), South China Sea Surface Current (SCSSC), and Kuroshio Current (KC) and its branch current (KBC). The hydrographic condition east of Taiwan is relatively simple and is controlled by the KC, a strong western boundary current that flows northward east of Taiwan year-round. In contrast, the marine environment west of Taiwan is strongly affected by the monsoon system [11], [12]. When the northeasterly monsoon prevails during the cold season, the cold, low saline, and nutrient-rich CCC flows southward along the coast of mainland China into the northern and central Taiwan Strait (TS); meanwhile, the warm and high saline KBC flows through the Luzon Strait and intrudes into the northern South China Sea (SCS) and the southeastern TS via the Penghu Channel [13], [14]. When the northeasterly monsoon wanes and the southwesterly monsoon begins during the warm season, the warm and low saline SCSSC, displacing the KBC, begins to penetrate northward into the northern TS [11], [15].

Siphonophores are common and worldwide, but in comparison with other zooplankton, they have often been poorly studied because their fragile body is easily broken by traditional sampling nets. Likewise, studies on community ecology and population distribution of siphonophores in the western North Pacific Ocean are also insufficient. A few surveys have been conducted in the East China Sea (ECS), SCS, Japanese waters, and the waters around Taiwan in recent times. For instance, on the northwestern continental shelf of the SCS, Li et al. [16] proposed that local coastal upwelling and surface ocean currents driven by the southwesterly monsoon increased the species number and abundance of siphonophores in summer; on the contrary, the northeasterly monsoon forced the cold coastal current into this area, resulting in low species richness and low abundance in winter. Li et al. [17] found out that 41 species of siphonophores in the northern SCS were more abundant in summer than in other seasons and aggregated in the nearshore region during the warm season and scattered in the offshore region during the cold season. Based on a large-scale survey in the ECS during 1 997–2000, Xu and Lin [18] noted that siphonophores were distributed mainly in southern and northern offshore areas, with water temperature, followed by salinity, as the main environmental factor to influence the distribution of siphonophore assemblages. In the nearshore waters of Japan, Kitamura et al. [19] found that *Muggiaea atlantica* was the most abundant taxon in early summer in the Osaka Bay and Tokyo Bay where were characterized by lower salinity. In the Sagami Bay, Grossmann and Lindsay [20] observed that the siphonophore communities could be related to the different water masses in the Bay, with an important influence of lateral transport of both tropical and subarctic species into the Bay by the different water masses. In the TS, it has been revealed that the distribution pattern of siphonophore assemblages was closely related to the hydrographic features, influenced by the dynamic nature of the currents in the area, with temperature, salinity, and zooplankton biomass being the three most important factors [21]. López-López et al. [22] found higher abundance of gelatinous carnivore zooplankton one month after the occurrence of a strong typhoon in northern Taiwan.

In recent years, there have been growing evidences that gelatinous blooms are increasing in frequency and persisting longer than usual [23], [24]. More studies have focused on the relationships between the oceanographic changes and siphonophore communities [25], [26], [27]. In the present study, our aim is to provide a comprehensive description of siphonophore diversity, distribution, and abundance, in conjunction with seasonal dynamics in the waters around Taiwan. Further, we explore the potential influence of environmental variables on the assemblage structure of siphonophores.

## Materials and Methods

### Ethics Statement

The study area is located between 21–26°N and 119–123°E (detailed location for each sampling station see Table S1). This study has been approved by the Taiwan Fisheries Research Institute. No specific permissions were required for the sampling locations and activities. The locations studied are not privately owned or protected in any way. The study did not involve any endangered or protected species.

### Field Sampling

**Figure 1:**
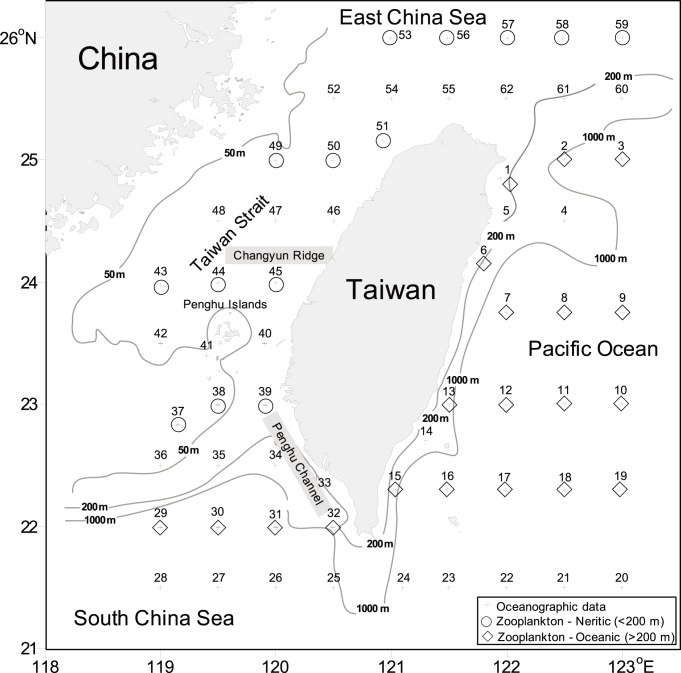
Map of Taiwan showing the locations of sampling stations.

Oceanographic data and zooplankton samples were obtained from four cruises of the RV *Fishery Researcher I*: 16–26 February (hereafter as February), 24 May to 3 June (hereafter as May), 4 August to 8 September (hereafter as August), and 4–14 November 2004 (hereafter as November). During each cruise, the oceanographic data, including temperature and salinity, were measured with a General Oceanics SeaBird CTD (SEB-911 Plus, Bellevue, Washington, USA) at 62 sampling stations (Figure 1). Water samples for chlorophyll *a* concentration measurements were also collected at 5, 25, 50, 75, 100, and 150 m depths using Go-Flo bottles (Havant, Hampshire, UK). Zooplankton samples were collected day and night at 34 of the 62 sampling stations (Figure 1) by vertical tows from a maximum depth of 200 m (or 10 m above the bottom at stations with a depth of <210 m) to the surface. Tows were made using an Ocean Research Institute (ORI) net with 330 µm mesh size and 1.6 m mouth diameter. The nets were towed at a mean speed of 1 m s^−1^ and were equipped with a Hydro-Bios mechanical flowmeter (Hydro-Bios, Kiel, Schleswing-Holstein, Germany) to calculate the volume of water filtered. Samples were preserved immediately on board in 5% borate-buffered seawater formalin.

### Preparation of Oceanographic and Biotic Data

Each zooplankton sample was divided into two subsamples with a Folsom splitter. Siphonophores were sorted from one stochastic subsample and identified and counted using a dissecting microscope. Because of their polymorphic structure and fragile nature, most siphonophores became fragmented in net samples, thus the numbers of nectophores or bracts of the physonects and hippopodiids were counted and then divided by ten to estimate their abundance. In addition, many calycophorans generally have two generations (polygastric and eudoxid phases) and each generation contains two distinct individuals (anterior and posterior nectophores of the polygastric phase, bract and gonophore of the eudoxid phase); therefore, nectophores (only anterior nectophores for diphyomorph calycophorans), bracts, and gonophores of each calycophoran species were counted separately and species abundance was calculated from the sum of the greater number of both generations (detailed methods to calculate the abundance of siphonophores and related citations see Table S2; [10], [28], [29], [30], [31], [32], [33], [34], [35]). The second subsample was repeatedly subdivided until the number of individual zooplankton in the last subsample was estimated to be 1000–2000 or fewer, and then the entire subsample was counted in order to calculate the overall abundance of zooplankton. Data were converted to the numbers of siphonophores (ind.) per 100 m^3^ and zooplankton per 1 m^3^ of water filtered by the net and presented as mean ± standard error (s.e.).

### Statistical Analysis

To describe the spatial variability of the assemblage structure of siphonophores, Shannon-Weaver diversity index (H’) [36] and Pielou’s index of evenness (J’) [37] were calculated for each station. Principal component analysis (PCA) [38] was used to characterize hydrographic regions and to distinguish temporal variability in temperature, salinity, and chlorophyll *a* data collected at each hydrographic station during the four cruises. In the study, except for the cruise in August 2004 that was carried out on and off (due to interruption of bad weather), the survey time of each cruise was 11 days. In order to demonstrate whether differences among seasons were significantly larger than those among replicate times of sampling within seasons, the survey time of each cruise was divided into 2 weeks randomly, each week was 5 or 6 days and included 31 (or 17 with zooplankton collection) stations, respectively. A 2-factor nested ANOVA [39], with seasons (4 levels) and weeks (2 levels) within seasons, was used to evaluate the differences of the environmental and biotic variables among seasons and among weeks within seasons. However, due to the convenience of sampling, the RV *Fishery Researcher I* generally collected the samples in the waters east of Taiwan first and the waters west of Taiwan later. The sampling time spent on the waters east and west of Taiwan was respectively about 5 days. Thus, we believe that in fact, the comparison among weeks within seasons also revealed the difference of the different sampling areas. This assumption was confirmed by Mann-Whitney *U* test [40] and the related results are shown in Table 1. In addition, in order to observe the spatio-temporal differences in the assemblage structure of siphonophores, multivariate statistics was performed with the PRIMER-6 software package. Similarity matrices of log(x+1)-transformed abundance of siphonophores in each sampling time were constructed using the Bray-Curtis Index [41]. These matrices were then employed to create plotting of classification diagrams of percentage similarity between samples using complete linkage. Meanwhile, non-metric multi-dimensional scaling (MDS) [42] was used to provide a two-dimensional visual representation of assemblage structure. The similarity percentage (SIMPER) routine showed the percentage contribution of each taxon to the average similarities within the different siphonophore assemblages [43]. In addition, relationships between abiotic (temperature and salinity) and biotic (chlorophyll *a* and zooplankton) matrices were explored through the BIOENV procedure by maximizing Spearman’s rank correlations (*r*_s_) between the similarity matrix (of Bray-Curtis similarity) of the abundance of siphonophores and the matrix (of Normalised Euclidean distance) of environmental similarities [44]. Finally, a one-way analysis of similarities (ANOSIM) [45] was applied to evaluate the effects of sampling time on the composition of siphonophore assemblage.

**Table 1:** Mann-Whitney *U* test and mean values of temperature, salinity, chlorophyll *a* concentration, zooplankton abundance, and abundance, species number, Shannon’s diversity, and Pielous’s evenness of siphonophore at different sampling areas (West: depth <200 m; East: depth >200 m) in the waters around Taiwan during the surveys.

	Sampling area			
	West	East	*U*	*p*
Temperature (°C)	25.07±0.45	26.69±0.24	1632	<0.01
Salinity	33.98±0.11	34.31±0.03	1657.5	<0.05
Chlorophyll *a* (µg l^−1^)	0.288±0.054	0.029±0.005	596	<0.001
Zooplankton (ind. m^−3^)	624±168	100±15	501	<0.001
Siphonophore (ind. 100 m^−3^)	898±171	257±17	955	<0.001
Species number	16±1	22±0	845	<0.001
Shannon’s diversity	2.76±0.11	3.39±0.03	1078	<0.001
Pielous’s evenness	0.70±0.02	0.77±0.01	1938	0.182

## Results

### Hydrographic Temporal Fluctuations

**Figure 2:**
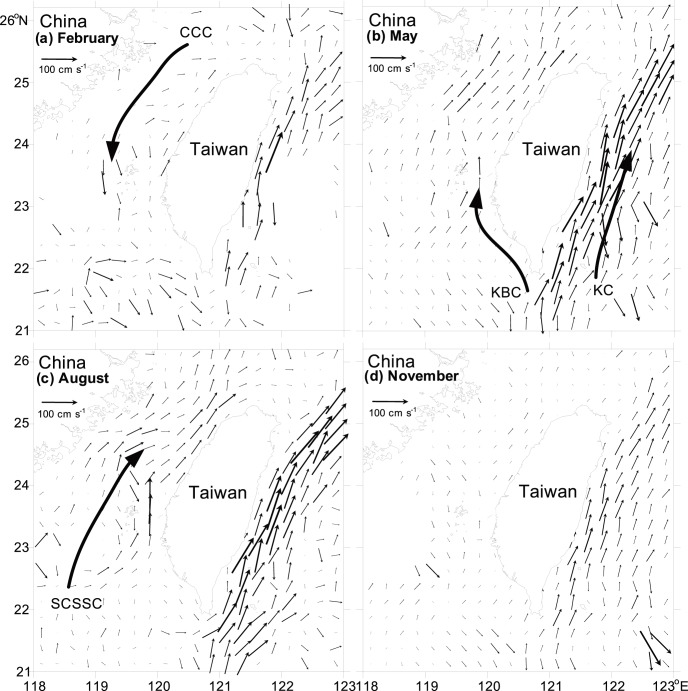
General circulation in the waters around Taiwan during the annual cycle in 2004. Data of seawater direction and velocity obtained from the Ocean Data Bank of the National Center for Ocean Research (NCOR), Taiwan.

**Figure 3:**
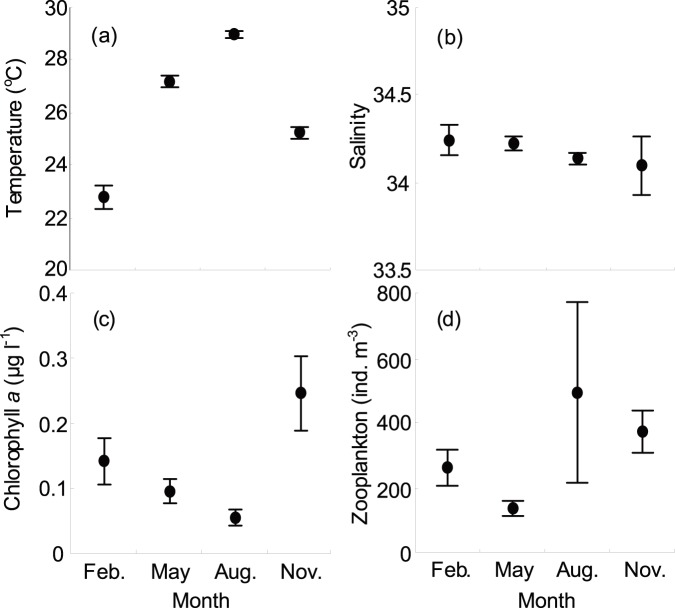
Mean values ( ± **s.e.) of hydrographic and biotic variables.** Plots show the mean values of temperature (a), salinity (b), chlorophyll *a* (c), and zooplankton (d) in the waters around Taiwan measured during the sampling period.

The maps of current direction and velocity of the waters around Taiwan in each cruise indicated the principal oceanographic features in the TS (data from the Ocean Data Bank of the National Center for Ocean Research, Taiwan; Figure 2). Throughout the study period, the surface temperature and salinity (at 10-m depth) fluctuated from 15.1°C to 30.2°C and from 31.0 to 34.7, respectively. Temperature showed significantly temporal and spatial differences (Table 2). Significantly higher temperature was observed in August than in February (nested ANOVA, *F = *11.448, *P*<0.05; Figure 3a), with a broader range in February (15.1–26.4°C) compared to the rest of the cruises; meanwhile, higher temperature was recorded in the waters east of Taiwan than in the waters west of Taiwan (nested ANOVA, *F = *19.829, *p*<0.001). Salinity gradually decreased from the highest values in February to the lowest in November (Figure 3b). Although no significant difference in salinity was detected between sampling times (nested ANOVA, *F = *0.061, *p* = 0.978), comparatively lower salinity was recorded in the waters west of Taiwan (nested ANOVA, *F = *10.694, *p*<0.001) (Table 2).

**Table 2:** Two-factor nested ANOVA (among Seasons and among Weeks within Seasons) of environmental and biotic variables and the abundance, species number, species diversity, and species evenness of siphonophores.

Source	df	MS	*F*	*p*	var. comp.	%
Temperature						
Seasons	3	424.6056	11.448	<0.05	6.250	67.52
Weeks(Seasons)	4	37.0885	19.829	<0.001	1.136	12.27
Residual	240	1.8704			1.870	20.21
Total	247					
Salinity						
Seasons	3	0.1540	0.061	0.978	–	–
Weeks(Seasons)	4	2.5438	10.694	<0.001	0.074	23.82
Residual	240	0.2379			0.238	76.18
Total	247					
Chlorophyll *a*						
Seasons	3	0.4237	0.371	0.779	–	–
Weeks(Seasons)	4	1.1413	19.743	<0.001	0.035	37.68
Residual	240	0.0578			0.058	62.32
Total	247					
Zooplankton						
Seasons	3	796068.4821	0.333	0.804	–	–
Weeks(Seasons)	4	2.39330E+06	3.572	<0.01	101369	13.14
Residual	128	670012.4280			670012	86.86
Total	135					
Abundance of siphonophores						
Seasons	3	185670.3235	0.063	0.977	–	–
Weeks(Seasons)	4	2.95612E+06	4.085	<0.01	131322	15.36
Residual	128	723641.0708			723641	84.64
Total	135					
Species number						
Seasons	3	225.4191	0.935	0.502	–	–
Weeks(Seasons)	4	241.0809	12.083	<0.001	13.008	39.47
Residual	128	19.9513			19.951	60.53
Total	135					
Species diversity						
Seasons	3	1.2466	0.484	0.711	–	–
Weeks(Seasons)	4	2.5757	7.054	<0.001	0.130	26.26
Residual	128	0.3652			0.365	73.74
Total	135					
Species evenness						
Seasons	3	0.0326	0.867	0.528	–	–
Weeks(Seasons)	4	0.0376	2.480	<0.05	0.001	8.01
Residual	128	0.0152			0.015	91.99
Total	135					

**Figure 4:**
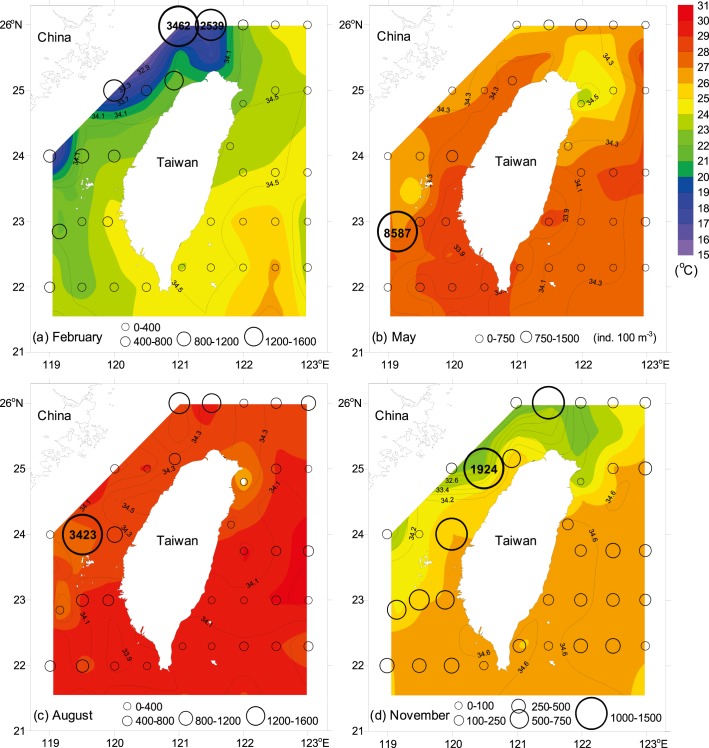
Contours of temperature, salinity, and siphonophore abundance. Color scale is the 10-m depth temperature, black line is the 10-m depth salinity, and circle is the total abundance of siphonophores.

The hydrographic characteristics of the waters around Taiwan show two typical patterns of summer (June–August) and winter (December–February) conditions. Between 23°N and 26°N, a strong temperature and salinity front was observed in the central TS during the northeasterly monsoon, reflecting two distinct water masses in the area (Figure 4a, 4d). Significantly lower temperature and salinity were found in the waters north of the Penghu Islands when the cold CCC flowed southward from mainland China. In contrast, a comparatively higher temperature and salinity water tongue was observed flowing northward along the southwestern coast of Taiwan. The isotherms displayed a northeast-southwest gradient, with the difference between these dense contours being up to 9°C. During the southwesterly monsoon, warmer waters (>27°C) were widely distributed over the study area, except comparatively low-temperature water observed in the Ilan Bay of northeastern Taiwan and the waters southwest of Penghu Islands (Figure 4b, 4c).

**Figure 5:**
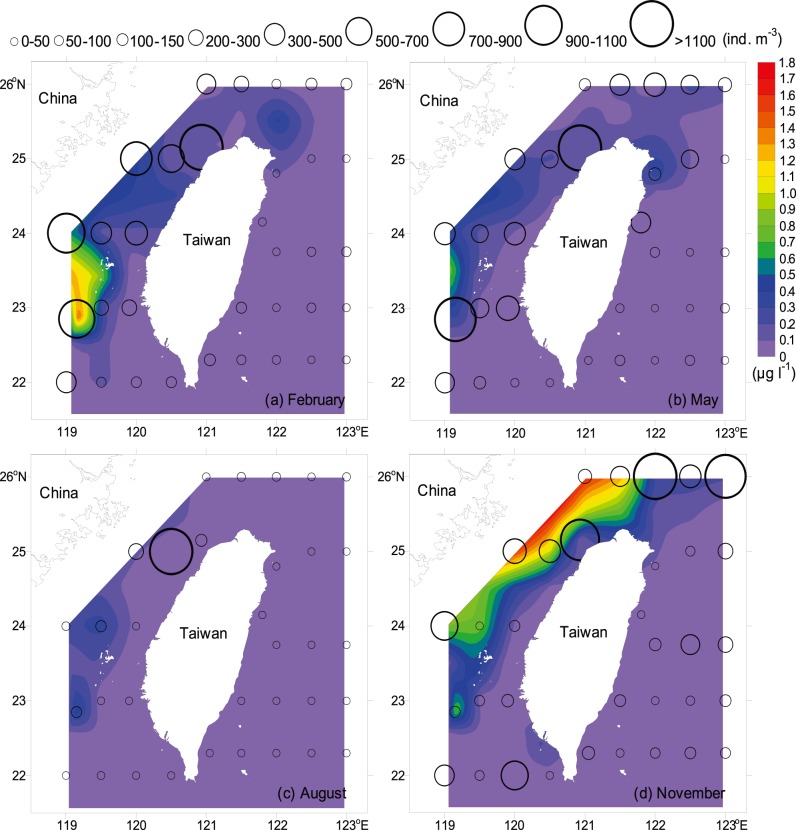
Horizontal distributions of chlorophyll *a* and zooplankton. Color scale is the average concentration of chlorophyll *a* in the upper 150 m and circle is the abundance of zooplankton.

The overall concentration of chlorophyll *a* (mean ± s.e.) was 0.135±0.018 µg l^−1^, ranging from 0.002 at Station 21 in May to 1.753 µg l^−1^ at Station 52 in November. Although no temporal difference in chlorophyll *a* concentration occurred in the study (nested ANOVA, *F = *0.371, *p* = 0.779; Table 2), higher mean concentration was recorded during the cold period than during the warm period (Figure 3c). Generally, chlorophyll *a* showed higher concentration in the waters west of Taiwan (nested ANOVA, *F = *19.743, *p*<0.001; Table 2), with the highest values in the waters west of Penghu Islands between 22.5°N and 24°N during the study period, except in autumn (Figure 5). Meanwhile, relatively higher concentrations of chlorophyll *a* were usually observed in the northern TS and lower concentrations in the waters southwest and east of Taiwan (<0.1 µg l^−1^).

Zooplankton abundance varied between 137 in May and 494 ind. m^−3^ in August, with an overall mean abundance (mean ± s.e.) of 316±77 ind. m^−3^. No significant temporal difference in zooplankton abundance was observed (nested ANOVA, *F* = 0.333, *p* = 0.804; Table 2, Figure 3d). Although zooplankton abundance did not have positive correlation with chlorophyll *a* concentration (Pearson’s correlation coefficient: *r* = 0.111, *n* = 136, *p* = 0.196; not shown), the distribution pattern of zooplankton abundance was rather similar to that of the chlorophyll *a* concentration, with apparently higher zooplankton abundances being generally recorded in the TS and the waters north of Taiwan (nested ANOVA, *F = *3.572, *p*<0.01; Table 2, Figure 5).

**Figure 6:**
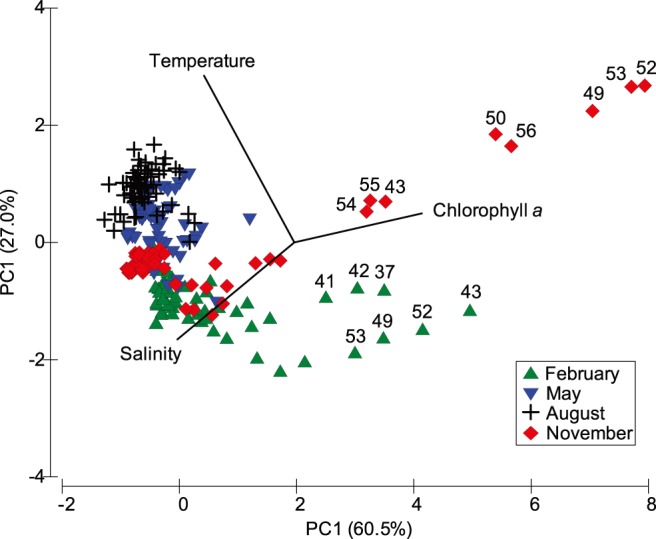
Plot of a principal component analysis (PCA). Diagram is established based on 10-m depth temperatures, 10-m depth salinity, and the average concentration of chlorophyll *a* in the upper 150 m.

The sampling stations during the survey were categorized into four temporal groups from the result of PCA of the three hydrographic variables, although with partial overlapping of stations (Figure 6). We noted that Stations 43, 49, 50 and 52–56 in November and Stations 37, 41–43, 49, 52 and 53 in February showed marked differences from the other stations, with higher chlorophyll *a* concentration and lower salinity.

### Abundance and Species Diversity of Siphonophores

**Figure 7:**
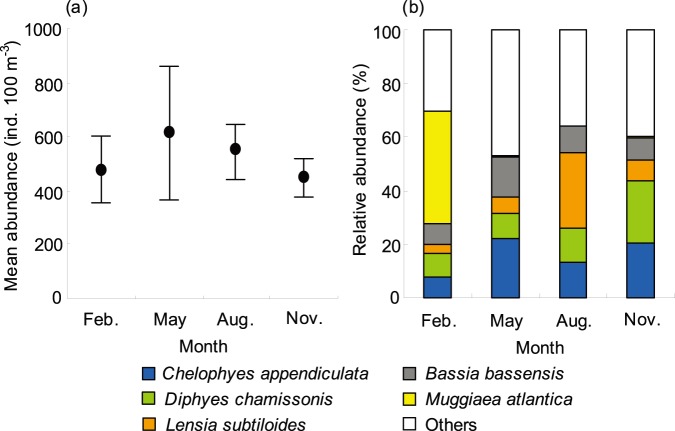
Temporal variation of siphonophore abundance and percentage contribution of the dominant species to total abundance. Blue is *Chelophyes appendiculata*, green is *Diphyes chamissonis*, orange is *Lensia subtiloides*, gray is *Bassia bassensis*, yellow is *Muggiaea atlantic*a, and white is other species.

The overall mean abundance of siphonophores (mean ± s.e.) during the four cruises was 521±76 ind. 100 m^−3^. Highest abundance was recorded in May (614±247 ind. 100 m^−3^) and lowest in November (450±71 ind. 100 m^−3^). There was no significant temporal difference in siphonophore abundance (nested ANOVA, *F* = 0.063, *p* = 0.977; Figure 7a), but apparently higher abundances were observed in the central and northern TS than in the waters east of Taiwan (nested ANOVA, *F* = 4.085, *p*<0.01; Table 2, Figure 4). Highest abundance was found in the waters southwest of Penghu Islands in spring, with a peak abundance of 8587 ind. 100 m^−3^ at Station 37 (Figure 4b) due to the high abundances of three dominant species *Chelophyes appendiculata*, *C. contorta*, and *Bassia bassensis*.

Although no temporal difference was observed in species number of siphonophores (nested ANOVA, *F* = 0.935, *p* = 0.502; Table 2), species number of siphonophores was more diverse in the warm period than in the cold period, ranging from 6 taxa at oceanic Station 29 in November to 35 taxa at Station 1 near coastal waters in August. In addition, the waters east of Taiwan showed significantly higher species number (nested ANOVA, *F* = 12.083, *p*<0.001; Table 2). The distribution patterns of the species diversity and species evenness of siphonophores were similar, both at the highest value in May. However, diversity (nested ANOVA, *F* = 0.484, *p* = 0.711) and evenness (nested ANOVA, *F* = 0.867, *p* = 0.528) of species also had no significant differences between sampling times (Table 2). The mean values (mean ± s.e.) of the two indexes fluctuated from 2.89±0.12 to 3.35±0.05 and from 0.70±0.02 to 0.78±0.01, respectively. The distributions in diversity and evenness of species of siphonophores showed a trend opposite to that of abundance, with higher values generally found in the waters south and east of Taiwan and lower in the waters north and west of Taiwan (nested ANOVA, *F* = 7.054, *p*<0.001 for diversity; nested ANOVA, *F* = 2.480, *p*<0.05 for evenness).

### Siphonophore Composition

In the present study, we identified 51 siphonophore taxa belonging to suborders Physonectae (3 families and 8 species) and Calycophorae (4 families and 43 taxa). The calycophoran family Diphyidae was the most diverse and dominant family (27 spp.) in this study, accounting for 79.5% of the total siphonophore numerical abundance, followed by the Family Abylidae (8 spp., 19.9%). Species compositions of siphonophores in each cruise are listed in Table 3. Significant temporal difference in siphonophore assemblage was evident by the ANOSIM analysis (one-way ANOSIM, Global *R* = 0.190, *p*<0.01; not shown), particularly between February and August (one-way ANOSIM, *R* = 0.352, *p*<0.01; not shown). Among the 51 taxa of siphonophores, 31 were recorded in all four cruises and 9 taxa were only collected in August. Species number of siphonophores was higher in August (49 spp.) than in the other sampling months (ranging from 35 to 39 spp.).

**Table 3:** Mean abundance (M, ind. 100 m^−3^) and frequency of occurrence (N, %) of siphonophore taxa identified in the collections of 2004.

Suborder/Family	Taxa	February		May		August		November	
		M	N	M	N	M	N	M	N
Physonectae									
Agalmatidae	*Agalma elegans*	0.07	38.2	0.35	29.4	0.17	38.2	0.09	29.4
	*Agalma okeni*	0.08	8.8	0.03	5.9	0.03	8.8	0.04	5.9
	*Cordagalma ordinata*					0.01	11.8		
	*Halistemma rubrum*	0.09	29.4	0.09	20.6	0.04	26.5	0.03	11.8
	*Lychnagalma utricularia*					0.002	2.9		
	*Nanomia bijuga*	0.89	82.4	4.30	67.6	0.86	91.2	1.97	85.3
Apolemiidae	Apolemiidae gen. sp.					0.003	2.9		
Physophoridae	*Physophora hydrostatica*					0.01	11.8	0.01	5.9
Calycophorae									
Abylidae	*Abyla haeckeli*	0.37	20.6	1.13	50.0	0.48	26.5	0.21	14.7
	*Abyla trigona*	0.09	8.8	0.06	2.9	0.02	2.9		
	*Abylopsis eschscholtzi*	20.16	94.1	28.04	100	27.87	94.1	44.00	97.1
	*Abylopsis tetragona*	33.49	97.1	18.92	94.1	8.61	88.2	6.81	76.5
	*Bassia bassensis*	35.69	100	91.26	100	54.56	94.1	38.16	79.4
	*Ceratocymba leuckarti*	0.74	38.2	2.69	50.0	0.28	17.6	0.47	17.6
	*Ceratocymba sagittata*	0.06	2.9	0.02	2.9	0.04	2.9		
	*Enneagonum hyalinum*			0.18	5.9	0.16	8.8	0.08	5.9
Diphyidae	*Chelophyes appendiculata*	36.81	100	134.23	100	71.49	100	91.39	100
	*Chelophyes contorta*	4.61	82.4	61.04	97.1	55.43	97.1	27.34	88.2
	*Diphyes bojani*	16.98	97.1	40.39	94.1	22.91	94.1	18.91	88.2
	*Diphyes chamissonis*	41.93	76.5	58.07	100	70.40	100	105.99	91.2
	*Diphyes dispar*	7.88	82.4	27.58	94.1	13.78	91.2	23.13	91.2
	*Eudoxoides mitra*	15.15	91.2	21.20	91.2	17.79	88.2	14.40	79.4
	*Eudoxoides spiralis*	21.71	91.2	27.17	97.1	9.63	88.2	20.22	79.4
	*Lensia ajax*					0.10	2.9		
	*Lensia campanella*	0.70	29.4	3.67	55.9	2.42	64.7	2.96	64.7
	*Lensia conoidea*	9.12	94.1	10.22	85.3	2.04	70.6	8.35	70.6
	*Lensia cossack*	2.22	50.0	4.39	61.04	1.01	38.2	0.83	29.4
	*Lensia exeter*					0.02	2.9		
	*Lensia fowleri*	0.12	8.8	0.07	8.8	0.74	35.3	0.10	5.9
	*Lensia grimaldi*					0.07	5.9		
	*Lensia hardy*	0.77	47.1	0.45	23.5	0.75	38.2	0.03	2.9
	*Lensia hotspur*	1.57	58.8	2.19	38.2	4.42	76.5	0.85	35.3
	*Lensia lelouveteau*					0.22	20.6		
	*Lensia meteori*	0.15	5.9			0.32	23.5		
	*Lensia multicristata*			2.06	32.4	0.05	5.9		
	*Lensia subtilis*	3.18	79.4	20.24	85.3	14.54	91.2	6.05	67.6
	*Lensia subtiloides*	17.13	79.4	37.60	97.1	153.01	100	33.75	67.6
	*Muggiaea atlantica*	200.32	29.4	3.21	14.7			0.32	8.8
	*Muggiaea kochi*			0.02	2.9				
	*Sulculeolaria chuni*	2.43	67.6	8.21	88.2	6.15	88.2	1.97	55.9
	*Sulculeolaria monoica*	0.12	5.9	0.02	2.9	0.12	8.8	0.02	2.9
	*Sulculeolaria quadrivalvis*	0.06	5.9	1.44	29.4	0.14	11.8	0.10	5.9
	*Sulculeolaria turgida*	0.59	32.4	3.07	61.8	2.11	79.4	0.63	26.5
Hippopodiidae	*Hippopodius hippopus*	0.15	47.1	0.08	38.2	0.07	38.2	0.05	20.6
	*Vogtia glabra*	0.14	44.1	0.08	32.4	0.10	55.9	0.08	32.4
	*Vogtia* sp.	0.005	2.9	0.004	2.9	0.03	23.5		
Prayidae	*Amphicaryon acaule*	0.11	5.9			0.05	2.9	0.04	2.9
	*Amphicaryon ernesti*			0.10	5.9	0.05	2.9		
	*Amphicaryon peltifera*					0.19	2.9		
	*Rosacea plicata*					0.14	23.5		
	*Praya* sp.	0.54	41.2	0.50	20.6	0.96	44.1	0.14	20.6
Total abundance		476		614		545		450	

The calycophorans *Chelophyes appendiculata*, *Diphyes chamissonis*, *Lensia subtiloides*, *Bassia bassensis*, and *Muggiaea atlantica* were overall the five most abundant species, accounting together for >61% of the total siphonophore numbers (Table 3). Except *M. atlantica* (occurrence rate only 13%), these species generally were present in >86% of all samples. Temporal changes in abundance were noted in some of the dominant species (Figure 7b). In general, the highest abundances of the dominant species (e.g. *C. appendiculata*) were found during the warm period. However, it was noted that some species were uniquely and significantly abundant in a specific sampling time. For instance, *M. atlantica* was the most abundant and collected almost exclusively in February; then, *L. subtiloides* was recorded in all cruises, but significantly higher abundances were observed in August, constituted 28.1% of the total siphonophores in August.

### Spatio-temporal Similarity of Siphonophore Assemblage

**Figure 8:**
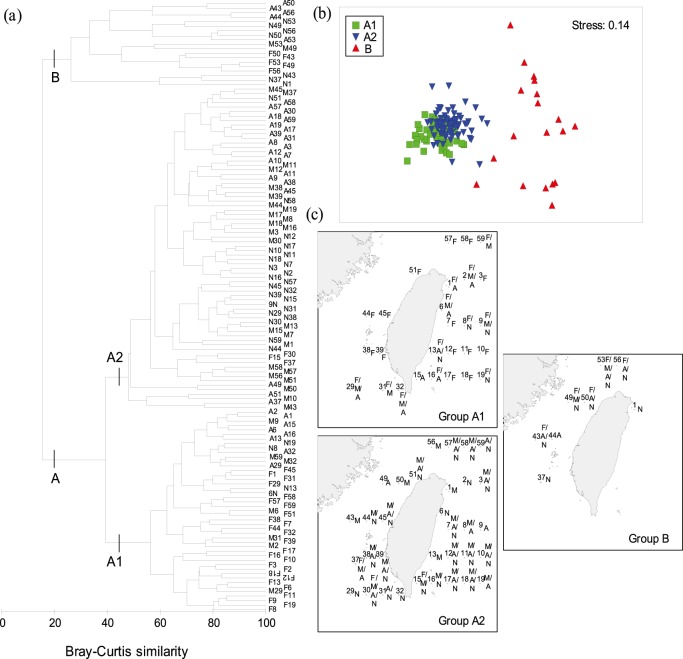
Dendrogram of Bray-Curtis similarity, multidimensional scaling (MDS) ordination, and geographic locations of the station groups. Classification diagram of percentage similarity between samples (a) is diagramed by the similarity matrices of log(x+1)-transformed abundance of siphonophores constructed using the Bray-Curtis Index. The MDS ordination of the station groups (b) based on Bray-Curtis similarity index provides a two-dimensional visual representation of assemblage structure. In addition, the geographic locations of the station groups (c) represented the sampling location and time (shown in the right side of stations) of stations within each station group. In these diagrams, F represents February, M represents May, A represents August, and N represents November. Green square is Group A1, blue inverted triangle is Group A2, and red triangle is Group B.

Cluster and ordination (MDS) analyses distinguished the species composition of siphonophores for all stations into two main groups of stations (A and B) by similarity level at 20% (Figure 8). Group A was further divided into two Subgroups of stations, namely A1 and A2. The characteristics of these groups and their environmental conditions are summarized in Table 4 and the species that contributed most (cutoff of the accumulated contributions of the species at 90%) to their structure are listed in Table 5.

**Table 4:** Mean values (± s.e.) of hydrographic, biotic, and siphonophore variables in the three station groups (according to Figure 8).

Group A1 (45 stations)			Group B (19 stations)		
Variables	Mean ± s.e.	RA	Variables	Mean ± s.e.	RA
Temperature (°C)	25.4±0.3	–	Temperature (°C)	23.2±1.0	–
Salinity	34.3±0.0	–	Salinity	33.4±0.3	–
Chlorophyll *a* (µg l^−1^)	0.04±0.01	–	Chlorophyll *a* (µg l^−1^)	0.50±0.12	–
Zooplankton (ind. m^−3^)	149±31	–	Zooplankton (ind. m^−3^)	924±472	–
Siphonophore (ind. 100 m^−3^)	261±33	–	Siphonophore (ind. 100 m^−3^)	1089±251	–
Species number	22±1	–	Species number	11±1	–
Shannon’s diversity	3.50±0.03	–	Shannon’s diversity	1.71±0.15	–
Pielous’s evenness	0.79±0.01	–	Pielous’s evenness	0.51±0.04	–
*Bassia bassensis*	41±4	15.8	*Muggiaea atlantica*	352±183	32.3
*Chelophyes appendiculata*	38±6	14.5	*Lensia subtiloides*	303±158	27.8
*Eudoxoides spiralis*	25±3	9.5	*Diphyes chamissonis*	300±104	27.6
*Abylopsis tetragona*	23±4	8.8	*Chelophyes appendiculata*	52±11	4.8
*Abylopsis eschscholtzi*	22±3	8.5	*Diphyes bojani*	15±4	1.4
Others (40 spp.)	112±26	42.9	Others (23 spp.)	67±26	6.1
**Group A2 (72 stations)**					
**Variables**	**Mean** ± **s.e.**	**RA**			
Temperature (°C)	27.1±0.2	–			
Salinity	34.3±0.0	–			
Chlorophyll *a* (µg l^−1^)	0.10±0.02	–			
Zooplankton (ind. m^−3^)	259±47	–			
Siphonophore (ind. 100 m^−3^)	534±119	–			
Species number	20±1	–			
Shannon’s diversity	3.27±0.03	–			
Pielous’s evenness	0.77±0.01	–			
*Chelophyes appendiculata*	120±34	22.5			
*Bassia bassensis*	76±16	14.2			
*Chelophyes contorta*	61±13	11.4			
*Diphyes chamissonis*	43±10	8.0			
*Abylopsis eschscholtzi*	41±5	7.6			
Others (48 spp.)	193±54	36.2			

RA: relative abundance (%) to total abundance.

**Table 5:** Discrimination of siphonophore species into three station groups (from Figure 8) based on the abundances of siphonophores by the SIMPER analysis.

Group A1 (Average similarity: 73.9%)			Group B (Average similarity: 52.2%)			
Species	MA	C	Species	MA		C
*Bassia bassensis*	41±4	13.1	*Diphyes chamissonis*	300±104		31.5
*Chelophyes appendiculata*	38±6	10.3	*Lensia subtiloides*	303±158		30.0
*Abylopsis eschscholtzi*	22±3	9.3	*Chelophyes appendiculata*	52±11		19.1
*Eudoxoides mitra*	19±3	9.0	*Diphyes bojani*	15±4		5.5
*Abylopsis tetragona*	23±4	8.9	*Muggiaea atlantica*	352±183		4.9
*Eudoxoides spiralis*	25±3	8.7	*Abylopsis eschscholtzi*	9±2		3.7
*Diphyes bojani*	15±2	7.3	*Lensia conoidea*	11±5		3.1
*Chelophyes contorta*	12±2	5.5	*Nanomia bijuga*	4±2		2.3
*Diphyes dispar*	9±2	4.4				
*Lensia conoidea*	6±1	4.2				
*Lensia subtilis*	7±1	3.8				
*Diphyes chamissonis*	14±5	3.7				
*Lensia subtiloides*	10±3	3.1				
Total	–	91.2	Total	–		91.1
**Group A2 (Average similarity: 73.1%)**						
**Species**	**MA**	**C**				
*Chelophyes appendiculata*	120±34	13.6				
*Bassia bassensis*	76±16	12.9				
*Chelophyes contorta*	61±13	11.3				
*Abylopsis eschscholtzi*	41±5	10.3				
*Diphyes bojani*	33±8	7.7				
*Diphyes dispar*	27±6	6.9				
*Diphyes chamissonis*	43±10	6.3				
*Eudoxoides mitra*	20±4	5.6				
*Eudoxoides spiralis*	21±4	5.0				
*Lensia subtiloides*	28±11	4.4				
*Abylopsis tetragona*	15±4	4.4				
*Lensia subtilis*	15±6	3.8				
Total	–	92.0				

MA: mean abundance (± s.e., ind. 100 m^−3^); C: percentage contribution (%) to within-group similarity.

Group A1 was comprised of 45 stations. This Group was clustered mainly by the stations sampled in February, although some stations of other seasons were also included. Group A1 was characterized by lowest chlorophyll *a* concentration and zooplankton and siphonophores abundances (Table 4). Forty-three siphonophore taxa were found in this Group, while *Bassia bassensis* and *Chelophyes appendiculata* were most abundant and important species, contributing 13.1% and 10.3% to the within-group similarity, respectively (Table 5). Within Group A1, the species with the highest contribution to similarity were *Abylopsis tetragona*, *A. eschscholtzi*, and *Eudoxoides mitra*, together contributing >27% to the within-group similarity.

Group A2 consisted of 72 stations mostly located in the southern TS and the waters east of Taiwan during May, August, and November. In total, 50 siphonophore taxa were found. Group A2 had a higher abundance of siphonophores than that of group A1, and was also dominated by *Chelophyes appendiculata* and *Bassia bassensis*, which representing 22.5% and 14.2% of the total catch, respectively (Table 4).

Group B contained 19 stations in the central and northern TS in all sampling times except Station 1 in November. This Group was characteristic by high abundance and low diversity of siphonophores and was associated with relatively lower temperature and salinity (Table 4). Only 27 siphonophore taxa were recognized and which were dominated by *Muggiaea atlantica*, *Lensia subtiloides*, and *Diphyes chamissonis*, responsible for 32.3%, 27.8%, and 27.6% of the Group, respectively. Among them *L. subtiloides* and *D. chamissonis* were important for this Group, both with a contribution >30% to the within-group similarity. On the contrary, the importance of *M. atlantica* was low, only contributing 4.9% to the within-group similarity (Table 5).

### Relation between Siphonophores and Hydrographic Variables

The BIOENV analysis evaluated the relationship between siphonophores and environmental variables (Table 6). Temperature, chlorophyll *a* concentration, and zooplankton abundance were the variables that best explained the pattern found in the structure of the siphonophore assemblages in the waters around Taiwan (Spearman’s rank correlation, *r*_s_ = 0.535, *p<*0.01). Besides, the single variable yielding the best rank correlation between matrices was chlorophyll *a* concentration (*r*_s_ = 0.502), implying that food source played an important role in the distribution of siphonophores.

**Table 6:** Correlation of siphonophores with environmental variables by use of the BIOENV routine.

Correlation with each variable	Correlation value
1 – Temperature	0.290
2 – Salinity	0.271
3 – Chlorophyll *a*	0.502
4 – Zooplankton	0.297
Best combination of one or more variables	1, 3, 4
	*r*_s_ = 0.535
	*p* = 0.01*

*Denotes result significant at <5%.

### Generation Succession of Predominant Siphonophore Species

The mean abundances of polygastric (asexual) and eudoxid (sexual) stages of the ten most abundant siphonophore species in different sampling months were shown in Table 7. Among these species, the eudoxids of *Chelophyes appendiculata* were significantly more numerous than the polygastrics in all sampling months. In contrast, in *Chelophyes contorta*, the abundance of eudoxid stage was very low, but density of polygastic stage was high during the warm period. Except the above two species, *Diphyes chamissonis*, *Bassia bassensis*, *Abylopsis eschscholtzi*, *D. bojani*, and *Eudoxoides spiralis* had slightly higher abundnaces of eudoxids than polygastrics; whereas, *Lensia subtiloides* and *D. dispar* had more abundnat polygastrics than eudoxids.

**Table 7:** Mean abundance (ind. 100 m^−3^) of asexual (polygastric, P) and sexual (eudoxid, E) stages of 10 predominant siphonophore species in different sampling month.

Species	Generation	February	May	August	November
*Chelophyes appendiculata*	P	0.74±0.32	10.04±9.85	0.05±0.04	3.93±1.32
	E	36.07±7.11	124.20±69.34	71.43±13.30	87.46±16.96
*Diphyes chamissonis*	P	12.06±4.70	25.60±10.25	26.98±13.42	12.79±6.08
	E	29.34±9.45	32.47±13.53	43.42±20.69	93.13±49.64
*Lensia subtiloides*	P	7.26±3.36	23.17±14.11	87.66±41.21	20.45±7.01
	E	9.87±4.40	14.43±8.82	65.35±52.69	13.30±5.91
*Bassia bassensis*	P	14.73±2.50	30.40±12.06	20.48±2.72	14.38±2.24
	E	20.96±3.44	60.86±21.48	34.08±4.08	23.78±3.60
*Muggiaea atlantica*	P	104.66±51.62	3.21±1.64	0±0	0.32±0.19
	E	95.66±55.76	0±0	0±0	0±0
*Chelophyes contorta*	P	4.61±0.89	59.32±25.72	32.64±6.38	27.34±3.65
	E	0±0	1.72±1.72	22.79±4.68	0±0
*Abylopsis eschscholtzi*	P	4.84±0.85	9.07±3.51	8.08±1.10	11.68±1.59
	E	15.32±3.79	18.96±4.82	19.79±2.72	32.33±4.14
*Diphyes bojani*	P	2.69±0.65	6.57±3.50	5.61±1.12	3.47±0.69
	E	14.29±2.77	33.86±13.46	17.31±2.73	15.44±2.34
*Eudoxoides spiralis*	P	5.61±1.13	7.01±2.26	2.70±1.07	5.50±1.51
	E	16.10±3.13	20.16±6.26	6.93±2.14	14.72±3.38
*Diphyes dispar*	P	7.07±2.21	19.45±9.31	13.41±1.82	15.60±3.27
	E	0.81±0.21	8.13±3.35	0.38±0.13	7.54±1.99

## Discussion

### Environmental Characteristics

The seasonal monsoon system and bathymetric topography are the two main physical parameters that affect the spatio-temporal variations in water masses and determine the through-flow transports, dominated alternately by the three currents, the CCC, KBC, and SCSSC, in the TS [11], [12]. are dominated alternately by three currents: the CCC, KBC, and SCSSC. During our study period, the CCC was predominant in winter from 26°N to the central TS, as evidenced by rapidly decreasing temperature and salinity from southeast to northwest. In the southeastern TS, water of relatively high temperature (>24°C) and salinity (>34) flowed northward through the Penghu Channel, signigfying the penetration of the KBC (Figure 2a, 4a). In contrast, there was an increase in northerly transport accompanied by a decrease in the westward intrusion of the KC through the Luzon Strait in summer, consequently the less-saline SCSSC replaced the KBC and widely distributed from south of the Penghu Islands to the northwestern part of the study area (Figure 2c, 4c). Compared to the TS, hydrographic conditions in the waters east of Taiwan are relatively stable. The KC predominated in the waters of eastern Taiwan, where temperature and salinity of surface waters remained >24°C and 34 year-round (Figure 4).

Chlorophyll *a* concentration and zooplankton abundance, in general, were lower in the waters east and south of Taiwan, and higher in the waters north of the Penghu Islands, particularly in the frontal zone and off northeastern Taiwan (Figure 5). Although how plankton respond to oceanic frontal systems is not clear, a few previous studies have indicated that the fronts would stimulate the productivity of plankton. For instance, in the Sea of Japan, highest fluorescence and copepod abundance were recorded near the frontal area [46]. Riemann et al. [47] reported a distinct increase in chlorophyll *a* associated with the thermal fronts bordering the subtropical convergence zone in the southern Sargasso Sea. Phytoplankton growth rates were near maximal in the subtropical convergence off New Zealand, but decreased to less than half of the maximal north and south of the convergence [48]. These are supported by our observation that the convergence of different water masses in the waters north of the Penghu Islands caused an elevated level of biological biomass and activity.

Our northern stations were located in the southern ECS where the KC flows through the area northeast of Taiwan. When the KC intrudes into the ESC shelf area in winter, a cold dome develops at the shelf break and forms a transition zone between the ECS and the KC [49]. This transition zone is characterized by an upwelling of nutrient-rich subsurface water to the surface and generally is highly productive [50]. Similar results were reported by Munk et al. [51] who found that high values of phytoplankton biomass and primary production were associated with a shelf break front and a dome of subsurface water between the Norwegian Coastal Current and the Jutland Coastal Current. However, during summer the prevailing southwesterly monsoon stops the surface intrusion of KC and in the meantime the frontal disturbance of the shelf edge area is replaced by topographic upwelling [52], [53], [54], [55]. The topographic upwelling provides the East China Sea shelf waters with a constant flux of nutrient-rich water [14], [54] and this upwelling has been considered a major source of nutrients for the shelf in summer [50], [54].

In addition, we found high chlorophyll *a* concentration and zooplankton abundance in the waters west of the Penghu Islands (Figure 5). When the KBC flows through the northern end of the Penghu Channel, where the northward current becomes faster and more turbulent when confronting with the narrower channel and shallower shelf, and finally is impeded by the Changyun Ridge [11], [12]. The deeper and colder subsurface water, when blocked by the shallower shelf and the Penghu Islands, rises and turns northwestward to the south of Penghu Islands and induces a cyclone (cold-core ring in the north hemisphere) because of the Ekman transport. The topographic upwelling enriches the nutrient and phytoplankton, and finally increased the abundance of zooplankton [56].

### Siphonophore Composition and Structural Assemblage

Currently, 175 valid siphonophore species, including 16 families and 65 genera, are recognized in the latest WoRMS world list [57]. Ninety-nine siphonophore species have been recorded in the western North Pacific Ocean [58], of which 51 taxa were present in this study (Table 3). The similarities of siphonophore composition between each cruise were >80% (not shown), indicating no significant temporal change in species composition. Siphonophores in the waters around Taiwan are mainly composed of a few common species occurring year-round (∼10 species, e.g. *Chelophyes appendiculata*, *Diphyes chamissonis*, and *Bassia bassensis*) and some occasional species only in August (e.g. *Cordagalma ordinate*, *Lychnagalma utricularia*, and *Lensia exeter*). Although there is little comparative information about geographic distribution on siphonophores in the waters around Taiwan, most species recorded in this study are tropic-subtropical [4], [25], [27], [59]. In general, the number of species found in different regions of the western North Pacific Ocean is low compared to 51 taxa in our study: 41 in the northern SCS [17], 38 on the northwest continental shelf of the SCS [16], 17 in the western waters of the TS [60], 26 in northern Taiwan [22], 41 in the ECS [18], and 5 species at the Yangtze River Estuary and its adjacent waters [61]. But, a higher species number (55 spp.) was observed from a transect off southern Taiwan between KC zone and SCS [62]. The increase in species number of siphonophores reported by the present study is a consequence of the major temporal and spatial scales considered.

*Chelophyes appendiculata*, *Diphyes chamissonis*, *Lensia subtiloides*, *Bassia bassensis*, and *Muggiaea atlantica* constituted the bulk of the siphonophore assemblage in the waters around Taiwan. Among these species, *M. atlantica* occurred exclusively in the waters north of the Penghu Islands in February, while the other four species distributed widely in the waters around Taiwan year-round. The high abundance of these dominant species is in agreement with reports from several adjacent areas, including the western waters of the TS [60], the ECS [18], and the Yangtze River Estuary and its adjacent waters [61]. Dominance of these species also was reported from other tropic-subtropical regions such as the Bay of Villefranche (northwestern Mediterranean) [27], [63], the east coast of South Africa [64], and the Gulf of Mexico [26], [30].

Our understanding of the role that seasonal succession of currents plays on siphonophore assemblage in the waters around Taiwan is still limited. Nevertheless, the contrasting hydrography of the waters around Taiwan, particularly in the TS, led us hypothesise that distinct assemblage of siphonophores reflects different hydrographic conditions. In the present study, cluster analysis suggests three groups of stations. Based on similarity indices, the species composition showed 9% (A1 versus A2), 22% (A1 versus B) and 29% (A2 versus B) differences (not shown) between each pair of these assemblages. A slight difference in species composition of siphonophores between the Groups A (A1 and A2) and B was noted. We found that nine taxa of siphonophores, namely *Cordagalma ordinate*, *Lychnagalma utricularia*, Apolemiidae sp., *Lensia ajax*, *L. exeter*, *L. grimaldi*, *L. lelouveteau*, *Amphicaryon peltifera*, and *Rosacea plicata*, were only recorded in August with very low abundance and frequency of occurrence (Table 3). It would be interesting to find out the cause for the above-mentioned difference between Groups A and B.

Divergence of near-surface water (upwelling) and sharp gradients of temperature and salinity (fronts) may accumulate or separate different assemblages of gelatinous zooplankton [4], [65]. Examinations of the species diversity and evenness of our sampling stations, we found that these indexes were similar in the waters southwest and east of Taiwan, mostly ranging between 3–4 for diversity and 0.7–0.9 for evenness. On the contrary, they varied temporally in the waters north of the Penghu Islands, particularly in the frontal area where the CCC meets the KBC (e.g. Stations 43, 49, 50, 53, and 56), showing significantly lower values during February and November and higher in May. We speculated that the decrease in species richness during February and November is probably due to the clogging of the sharp frontal gradients and further constrained by bottom topography. On the other hand, when the northeasterly monsoon weakens in May, there is apparently a greater northward flow of the KBC (Figure 2), consequently, strengthening the northward transport of siphonophores from southern to northern TS.

The Groups A1 and A2 are confined mainly to the waters southwest and east of Taiwan associated with the KBC and KC. The areas naturally have low temporal oscillations in temperature and salinity and the highest mean diversity and species number of siphonophores. The two Groups were basically characterized by the dominance of *Chelophyes appendiculata* and *Bassia bassensis*, which were the top two most abundant and common siphonophores in the study. But other predominant species were different between the Groups, *Eudoxoides spiralis* and *Abylopsis tetragona* for the Group A1 and *C. contorta* and *Diphyes chamissonis* for the Group A2 (Table 4, 5).

*Chelophyes appendiculata* is among the most common species of calycophoran siphonophores inhabiting in the upper layers [33], [35]. Previous studies have reported that its peak abundances occurred in spring and autumn in the Bay of Villefranche [63] and during October in the southern Gulf of Mexico [26]. Similarly, in the Nanwan Bay of southern Taiwan Zhang et al. [66] found that *C. appendiculata* was the predominant species in late autumn and was substantiated by our similar result. In addition, we noted that the highest abundance of *C. appendiculata* was recorded at Station 37 in May when salinity was relatively low. According to Gibbons and Thibault-Botha [67], *C. appendiculata* is widely distributed in the oceanic realm and also in the near-shore waters around southern Africa. Furthermore, Sanvicente-Añorve et al. [26] observed abundance of *C. appendiculata* over the middle and outer shelves of the southern Gulf of Mexico, with salinities between 30.7 and 37.0. These results indicated that this species appears to be highly tolerant to a wide range of salinity.

The highest abundance of *Bassia bassensis* was observed at Station 37 in May when chlorophyll *a* concentration and zooplankton abundance were high. This species was generally abundant and frequent in the study area, in contrast to its very low or zero occurrence at some neritic stations where temperature was below 23°C. *Bassia bassensis* is a common epipelagic calycophoran which mainly occurs in the top 50 m in temperate waters of the three main oceans and the Mediterranean Sea [33], [65]. Studying neritic and oceanic waters of the southern Gulf of Mexico, Gasca [30] found *B. bassensis* among the most abundant siphonophores and mainly at the 20–80 m stratum [26]. Off the coast of Chile, Pagès et al. [4] reported that *B. bassensis* is most abundant at depths shallower than ∼50 m, especially in oceanic waters with temperature >19°C. Similarly, this species was the most abundant siphonophore in surface waters adjacent to the Easter Island where temperature was ∼21°C [25], [68].

Group B is composed mainly of species distributed in neritic waters. This zone is confined to depths <100 m, with great variations in temperature and salinity due to the penetration of the CCC. The area was also characterized by an ample supply of food, as indicated by the highest chlorophyll *a* concentration and zooplankton abundance observed in the study (Table 4).

*Muggiaea atlantica*, a common component in the coastal, cool, and productive waters, such as the Bay of Villefranche [27], Benguela Current [65], Agulhas Current [64], and Chiloé Interior Sea [69], was the most abundant and important species in Group B. The density of *M. atlantica* may reach up to 140 ind. 100 m^−3^ in Friday Harbor [8] and 239 ind. 100 m^−3^ in the Humboldt Current system [4], as well as our findings with a peak mean abundance of 200 ind. 100 m^−3^ in February (Table 3). Along the east coast of South Africa Thibault-Botha et al. [64] reported that *M. atlantica* nearly completely dominated the inner stations to the extreme south of their study area with temperature varying between 16–19°C. A similar result has also been reported by Batistić et al. [70] who pointed out that temperature of about 14–18°C is optimal for the reproduction of *M. atlantica* in the marine Mljet lakes (Adriatic Sea). Likewise, in the present study, we noted high abundances of *M. atlantica* at Stations 49, 53 and 56 in February when temperature was only 16–18°C. We speculated that the high abundance of *M. atlantica* in our study probably was resulted from enhanced population growth favored by lower temperature. This phenomenon further suggests that this species is more favorable to low temperature environment than other tropical siphonophores.

*Diphyes chamissonis* and *Lensia subtiloides* are largely neritic and warm-water species and distributed mainly in the Indo-Pacific waters [35], [71]. *Diphyes chamissonis* and *L. subtiloides*, accounting for 24.8% of the total siphonophore abundance in our study, showed the temporal peak abundance in November and August, respectively (Table 3). They were widespread in the waters around Taiwan but more common in the neritic waters north of the Penghu Islands. Our result indicated that *D. chamissonis* and *L. subtiloides* are probably good indicator species for neritic waters. During a survey of the ECS Xu and Lin [18] found an autumn aggregation of *D. chamissonis* in the Yangtze River estuary. Along the east coast of South Africa *D. chamissonis* was also abundant in autumn and rare or totally absent in spring and summer [64]. Consistent result was present in our study, with the highest abundance at Station 50 in November when temperature and salinity were below 23°C and 32, respectively. Compared with *D. chamissonis*, *L. subtiloides* showed significantly higher abundance in August than in other three sampling times (Table 3), with the peak abundance at Station 56 off the northern Taiwan. According to previous studies, *L. subtiloides* is usually rare or absent in the Gulf of Mexico [30], [59], eastern South Pacific Ocean [4], [25], [66] and Adriatic Sea [72], but not in the coastal waters east of South Africa [64]. The distribution of *L. subtiloides* in the present study indicates that it is able to tolerate a wide range of temperatures and relatively higher abundant in temperature >26°C.

### Factors Affecting the Distribution of Siphonophores

The seasonal distribution and abundance of planktonic cnidarians is primarily governed by the factors controlling their reproductive cycle [7]. Some gelatinous species, including siphonophores, have rapid asexual reproductive processes and their populations respond rapidly to favorable environmental conditions [5]. Arai [6] suggested that the release of medusa from hydroids can be affected by several factors, such as temperature, salinity, food abundance, or the light/dark ratio. In the present study, temperature, chlorophyll *a* concentration, and zooplankton abundance were identified as the three major environmental factors to influence the distributional pattern of the siphonophore assemblages (Table 7). This result coincided with Gibbons and Richardson [73] who found that seasonal and inter-annual variability on jellyfish peaks in the North Atlantic Ocean can be related to peaks in phytoplankton and zooplankton abundance and peaks in temperature changes.

The role of temperature may be more complex because it can directly or indirectly regulate the marine food web through the alteration of the bottom-up or top-down controls [74], [75]. In general, most jellyfish species have the potential to bloom during the warm season (spring or summer) in temperate regions [76]. An increase in temperature could enable greater spring survival of young medusa, faster individual growth rates, and overall jellyfish biomass [77]. In the Bay of Villefranche, Licandro et al. [27] found that higher temperature had a positive influence on the siphonophore abundance. Hosia and Båmstedt [10] also reported that the locally higher densities of *Dimophyes arctica* and *Lensia conoidea* during the warm season in Norwegian Korsfjord were probably due to higher temperature. Our study (Figure 7a) is in agreement with previous findings of a temporal maximum in siphonophore abundance in May [27], [69], [78]. Distinctly higher abundances in several predominant species, particularly *Chelophyes appendiculata* and *L. subtiloides*, were observed in high temperature (>26°C) in May or August. Nevertheless, in the southern Gulf of Mexico, Sanvicente-Añorve et al. [26] noted that temperature higher than 28.1°C depressed most siphonophore populations. Similarly, Lo et al. [62] found that the abundance of siphonophores in the waters around Taiwan was notably reduced when temperature was higher than 28.5°C.

In the study area, significantly higher chlorophyll *a* concentrations were recorded in the northern half of the TS (the frontal area that introduces nutrients via the CCC) and the waters west of the Penghu Islands (an area of topographic upwelling due to the KBC obstructed by the Changyun Ridge) (Figure 6). It is well known that siphonophores are carnivorous zooplankton, consuming mainly copepods, the major constituent of the zooplankton community [1], [79]. Phytoplankton growing in frontal or mixed areas may provide a better food source for zooplankton [80]. In the present study, we found that the distribution pattern of zooplankton was parallel to the chlorophyll *a* concentration. Meanwhile, the higher abundances of siphonophores were found very closely related to the two areas with higher zooplankton abundance. In the NW Mediterranean Sea, Sabatés et al. [81] reported that high densities of coastal and offshore species of siphonophores were found close to the shelf/slope front, and could be related to increased primary and secondary productions in the frontal area. Li et al. [16] proposed that copepod abundance appeared to be the most significant factor to influence the distribution and abundance of nearshore siphonophores in the northwestern SCS. These studies led us believe that the higher siphonophore abundances would be correlated with the elevated primary and secondary productivity.

For calycophoran siphonophores, the breeding season was determined by successions between higher abundances of the asexual (polygastric) and sexual (eudoxid) stages [63]. In the present study, we noted that the high abundances of *Chelophyes appendiculata*, with eudoxids 19 times more numerous than polygastrics, were recorded during the warm period (Table 7) and corresponded to higher zooplankton abundances. It was worth noted what factor caused the difference in the amounts of polygastric and eudoxid. However, knowledge regarding the life history and reproductive capacity of siphonophores is scarce. Comparing the speed of maturation of *Muggiaea kochi* under different temperature conditions, Carré and Carré [82] found that at 18°C the eudoxids were liberated between day 12 and 14, then eudoxids produced mature gonophore-releasing gametes from day 19; whereas, at 24°C eudoxids were liberated between days 10 and 11 and began to release gametes from day 15. At Friday Harbor Purcell [8] observed that the production and maturation times of the eudoxids of *Muggiaea atlantica* increased with prey availability. Hosia and Båmstedt [10] suggested that favourable prey concentrations contributed to the higher densities of eudoxid stage of *Lensia conoidea* and *Dimophyes arctica* in summer and autumn in Norwegian fjords. In the Boka Kotorska, Pestorić et al. [78] found a significant positive correlation between *M. atlantica* eudoxids and their potential prey and proposed that a rapid reproductive response of this siphonophore to its potential prey densities. These reports seemed to indicate that higher temperature and seasonality in zooplankton biomass are the control mechanisms of seasonal cycles of the major gelatinous predators, and consequently, affect their abundance.

In addition to the above-mentioned variables, salinity is usually an important factor structuring the siphonophore assemblages. Abrupt changes in salinity may affect the buoyancy, reproduction, and prey consumption rate of cnidarians [83], [84]. According to the analysis of long-term records collected in the northwestern Mediterranean [27] and in other regions worldwide [85], it seems to suggest that salinity gradients may significantly affect the abundance of different jellyfishes. In the Bay of Villefranche, Licandro et al. [27] found that the abundance of the most dominant calycophoran siphonophores, in particular, *Muggiaea kochi*, *Chelophyes appendiculata*, and *Abylopsis tetragona*, significantly increased under different salinity optima. In the Mondego estuary, salinity was the main factor affecting jellyfish assemblages, explaining around 20% of the variability observed during summer, being particularly related to siphonophore abundance [86]. Sanvicente-Añorve et al. [59] also suggested that even extreme salinity values (>36.5 or <34) might depress siphonophore populations in the coastal area of the southern Mexican Gulf. However, our result is quite different from the above-mentioned studies. No significant correlation between salinity and siphonophore assemblage was observed during our investigation.

In conclusions, no significant temporal difference in siphonophore abundance was found in the present study. In contrast, the composition and distribution of siphonophore assemblage showed temporal and spatial differences. More diverse siphonophores were observed during the warm period and in the waters southwest and east of Taiwan. The monsoon-driven dynamics of the CCC, SCSSC, and KBC in the study area play an important role on the transportation of siphonophores. The distribution of siphonophore assemblage was heavily influenced by the different hydrographic features, with temperature, chlorophyll *a* concentration, and zooplankton abundance as the three most important variables.

## Supporting Information

Table S1 Detailed location and depth of 62 sampling stations.
(DOC)

Table S2 Methods of calculating the abundance of siphonophores and citations. The abundance of siphonophores of the different suborders or families is respectively estimated based on their polymorphic structure.
(DOC)
