# Supplementary material for: Hydrographic Processes Driven by Seasonal Monsoon System Affect Siphonophore Assemblages in Tropical-Subtropical Waters (Western North Pacific Ocean)
Source: PLoS One. 2014 Jun 16;9(6):e100085. doi: 10.1371/journal.pone.0100085 (PMC4059725; doi:10.1371/journal.pone.0100085)
Supplement: Table S1 — Detailed location and depth of 62 sampling stations. (DOC) [file pone.0100085.s001.doc]

| Station | Latitude (N) | Longitude (E) | Depth | Station | Latitude (N) | Longitude (E) | Depth |
| --- | --- | --- | --- | --- | --- | --- | --- |
| 1 | 24°51.484 | 121°59.429 | 313 | 32 | 22°00.264 | 120°29.848 | 314 |
| 2 | 25°00.194 | 122°29.840 | 1457 | 33 | 22°23.732 | 120°19.695 | 145 |
| 3 | 25°00.934 | 123°00.477 | 1607 | 34 | 22°30.481 | 120°00.453 | 592 |
| 4 | 24°30.021 | 122°30.292 | 579 | 35 | 22°29.516 | 119°30.510 | 236 |
| 5 | 24°29.823 | 122°00.467 | 628 | 36 | 22°29.211 | 118°59.683 | 92 |
| 6 | 23°59.447 | 121°40.777 | 219 | 37 | 22°55.601 | 119°06.020 | 27 |
| 7 | 23°46.273 | 122°00.381 | 3414 | 38 | 23°00.161 | 119°29.664 | 81 |
| 8 | 23°45.322 | 122°30.579 | 3055 | 39 | 23°00.405 | 119°54.751 | 129 |
| 9 | 23°45.520 | 123°00.149 | 3656 | 40 | 23°29.928 | 119°55.012 | 104 |
| 10 | 23°00.416 | 123°00.591 | 5744 | 41 | 23°25.995 | 119°29.593 | 60 |
| 11 | 23°01.167 | 122°30.209 | 5540 | 42 | 23°30.177 | 119°00.028 | 58 |
| 12 | 23°01.250 | 122°01.173 | 4896 | 43 | 24°00.286 | 118°59.994 | 63 |
| 13 | 23°00.828 | 121°30.085 | 2054 | 44 | 24°00.221 | 119°29.973 | 66 |
| 14 | 22°40.245 | 121°15.061 | 1226 | 45 | 24°00.435 | 120°00.204 | 46 |
| 15 | 22°15.512 | 121°00.088 | 1212 | 46 | 24°30.205 | 120°29.694 | 56 |
| 16 | 22°15.623 | 121°30.544 | 843 | 47 | 24°29.701 | 120°00.143 | 64 |
| 17 | 22°15.497 | 122°00.723 | 4608 | 48 | 24°30.162 | 119°30.256 | 68 |
| 18 | 22°15.698 | 122°30.532 | 4844 | 49 | 24°59.919 | 119°59.918 | 57 |
| 19 | 22°15.736 | 123°00.169 | 3655 | 50 | 25°00.000 | 120°29.800 | 79 |
| 20 | 21°30.454 | 122°59.497 | 5035 | 51 | 25°05.013 | 120°54.572 | 75 |
| 21 | 21°30.163 | 122°29.646 | 4787 | 52 | 25°30.710 | 120°30.179 | 68 |
| 22 | 21°30.701 | 122°00.263 | 3318 | 53 | 25°59.947 | 120°59.684 | 84 |
| 23 | 21°29.686 | 121°29.905 | 2083 | 54 | 25°29.838 | 121°00.095 | 95 |
| 24 | 21°30.552 | 121°00.506 | 1022 | 55 | 25°30.151 | 121°30.362 | 124 |
| 25 | 21°29.651 | 120°29.902 | 1838 | 56 | 25°59.400 | 121°30.029 | 73 |
| 26 | 21°30.725 | 119°59.809 | 2931 | 57 | 26°00.450 | 121°59.954 | 104 |
| 27 | 21°30.210 | 119°29.785 | 2989 | 58 | 26°00.106 | 122°30.150 | 110 |
| 28 | 21°30.664 | 118°59.623 | 2813 | 59 | 26°00.115 | 123°00.423 | 99 |
| 29 | 22°01.674 | 119°00.734 | 1790 | 60 | 25°30.417 | 123°00.104 | 741 |
| 30 | 22°00.809 | 119°30.159 | 2425 | 61 | 25°30.137 | 122°29.896 | 334 |
| 31 | 21°59.691 | 120°00.591 | 1201 | 62 | 25°30.015 | 121°59.862 | 123 |

**Table S1:** Detailed location and depth of 62 sampling stations.
