# Supplementary material for: Hydrographic Processes Driven by Seasonal Monsoon System Affect Siphonophore Assemblages in Tropical-Subtropical Waters (Western North Pacific Ocean)
Source: PLoS One. 2014 Jun 16;9(6):e100085. doi: 10.1371/journal.pone.0100085 (PMC4059725; doi:10.1371/journal.pone.0100085)
Supplement: Table S2 — Methods of calculating the abundance of siphonophores and citations. The abundance of siphonophores of the different suborders or families is respectively estimated based on their polymorphic structure. (DOC) [file pone.0100085.s002.doc]

**Table S2:** Methods of calculating the abundance of siphonophores and related citations.

| Suborder/Family | Methods of calculating abundance | Citations |
| --- | --- | --- |
| Physonectae  Agalmatidae  Apolemiidae  Physophoridae | If the pneumatophore is present, it was counted as one colony. Or, if the pneumatophore is absent, the numbers of nectophores or bracts were counted and then divided by ten as the number of their abundance, if the number was less than ten, it was counted as one colony. | [10], [28], [29], [30], [31], [32], [33], [34], [35] |
| Calycophorae  Abylidae  Diphyidae | Anterior and posterior nectophores of the polygastric phase and bract and gonophore of the eudoxid phase were counted separately and species abundance was calculated from the sum of the greater number of both generations. |
| Hippopodiidae | Numbers of nectophores were counted and then divided by ten as their number of abundance, if the number was less than ten, it was recorded as one colony. |
| Prayidae | Nectophore of the polygastric phase and bract and gonophore of the eudoxid phase were counted separately and species abundance was calculated from the sum of the greater number of both generations. |
